# Vitreoretinal Surgery for Intraocular Complications Following Radiotherapy Treatment of Uveal Melanoma

**DOI:** 10.3390/cancers18010095

**Published:** 2025-12-27

**Authors:** Thomas Joseph Padley, Rumana Hussain, Antonio Eleuteri, Hung-Da Chou, Carl Groenewald, Heinrich Heimann

**Affiliations:** 1 Liverpool Ocular Oncology Centre, Prescott Street, Liverpool L7 8XP, UK; carl.groenewald@liverpoolft.nhs.uk (C.G.); heinrich.heimann@liverpoolft.nhs.uk (H.H.); 2 Research & Innovation Department, NHS University Hospitals of Liverpool Group, Edwards Building, Liverpool L7 8YE, UK; antonio.eleuteri@liverpoolft.nhs.uk; 3 Liverpool Ocular Oncology Research Group, Department of Eye and Vision Science, Institute of Life Course and Medical Sciences (ILCaMS), University of Liverpool, Liverpool L7 8TX, UK; hung-da.chou@liverpoolft.nhs.uk

**Keywords:** uveal melanoma, vitrectomy, radiotherapy

## Abstract

(1) Background: Globe-sparing radiotherapy is widely utilised in the treatment of uveal melanoma, but often results in complications requiring vitreoretinal intervention. The outcomes of secondary vitrectomy remain unclear. A multidisciplinary approach involving vitreoretinal and ocular oncology specialists is essential to managing complications. (2) Methods: We reviewed 1794 patients treated with radiotherapy for uveal melanoma between 2012 and 2022. In total, 70 patients underwent secondary vitrectomy after primary radiotherapy treatment. The outcomes included overall tumour control and visual outcome. (3) Results: Complications requiring vitrectomy were more common after proton-beam radiotherapy than plaque brachytherapy (5.4% versus 3.0%). Common indications included vitreous haemorrhage (39%) and retinal detachment/toxic tumour syndrome (31%). The affected tumours were larger, more often ciliary body in origin, and associated with a worse prognosis. Vitrectomy patients had higher rates of enucleation (9% versus 3%), metastasis (16% versus 6%), and visual decline (average 0.60 LogMAR), with limited visual improvement (≥3-line gain in 13%). Proton-beam patients had worse outcomes than plaque brachytherapy patients. (4) Conclusions: Vitreoretinal complications after uveal melanoma radiotherapy are rare, but timely treatment by those with experience may enable patients to keep their eye in situations where enucleation would be the only alternative. Patients and clinicians must understand the risks of complications to make informed decisions about treatment plans, with vitreoretinal surgeons and ocular oncologists key to outcomes.

## Simple Summary

Uveal melanoma is a rare eye cancer that is commonly treated with radiotherapy, as it provides an opportunity for treatment while allowing patients to keep their eye rather than having it surgically removed. This treatment can, however, lead to complications needing surgery, often a procedure called a vitrectomy. In this study, we analysed patients who underwent vitrectomy after radiotherapy to better understand future visual quality and the degree of tumour control. We found that these patients often had aggressive cancers and were more likely to suffer a decline in vision, exhibit a lack of tumour control, and more commonly require removal of the affected eye. The findings highlight the importance of collaborative management between oncologists and eye surgeons. This data allows both doctors and patient to understand the possible risks before starting radiotherapy treatment.

## 1. Introduction

Uveal melanomas are very rare intraocular malignant tumours with a reported incidence between 5.3 and 10.9 cases per million in Caucasian populations [1,2,3]. The mainstay globe-sparing treatments of uveal melanoma include plaque brachytherapy, external radiotherapy sources (protons, stereotactic, and gamma knife), and surgical resection [4,5,6], offering local tumour control without increasing metastatic potential. These therapies are favoured as they provide an opportunity to save vision, avoiding the physical and psychological complications of enucleation, and demonstrate similar efficacy and survival rates compared to enucleation [4,7,8], although enucleation remains a pertinent treatment for large or posteriorly located tumours. However, these procedures carry risks of post-operative vitreoretinal complications including vitreous haemorrhage, retinal detachment, epiretinal membrane formation, neovascular complications leading to rubeotic glaucoma, and macular hole [9,10,11,12,13]. With a worldwide tendency to reduce the number of enucleations, prioritising eye preservation, and the increasing use of tumour biopsies, a corresponding rise in vitreoretinal complications is to be expected [14]. Consequently, vitreoretinal surgeons are likely to become increasingly involved in the management of more challenging cases of uveal melanoma in the near future.

Plaque brachytherapy is the preferred treatment where possible for small–medium-sized tumours in the periphery or mid-periphery; however, proton-beam radiotherapy is the standard in the United Kingdom for tumours at the posterior pole and for tumours too large for plaque brachytherapy [7,15,16]. Vitreoretinal surgeries may be performed as part of initial melanoma treatment, either in conjunction with tumour resection or as an adjunct to other therapies. Treatment combinations can involve vitrectomy with plaque radiotherapy and silicone oil shielding to reduce the degree of radiation-related side effects [17], with surgical endoresection [18], or when there is a co-existing ocular disorder such as retinal detachment or vitreous haemorrhage [19]. The safety and efficacy of vitrectomy regarding potential for tumour dissemination including metastasis and local recurrence has previously been theorised, although the data currently suggests that this is safe to perform [19,20,21].

Secondary vitrectomy is performed after primary melanoma treatment, often because complications have developed; these include but are not limited to vitreous haemorrhage, retinal detachment, and epiretinal membrane [20,22,23,24]. Vitrectomies are performed with three goals in mind; improving visual outcomes, avoiding secondary enucleation, or providing a better view for tumour monitoring. However, there have been concerns in the past of local tumour seeding and increased metastatic risk from vitreoretinal surgery following tumour treatment, but this has not been referenced in the literature [20,25]. There is also particular importance placed on avoiding tumour-affected areas when inserting trocars. As a result, many vitreoretinal surgeons not associated with Ocular Oncology Centres are often reluctant to proceed with necessary surgery and prefer to re-refer patients to such centres, often significantly delaying the necessary treatment. There are few studies available that investigate the safety of secondary vitrectomy in eyes containing treated uveal melanoma, and so data is limited on this topic [20].

The Liverpool Ocular Oncology Centre (LOOC) is one of three centres in England treating uveal melanoma and runs under the care of three ocular oncologists and vitreoretinal surgeons. This study aims to assess the incidence, indications, and outcomes of vitreoretinal interventions following radiotherapy treatment of uveal melanoma.

## 2. Materials and Methods

The LOOC maintains a database of all patients diagnosed and treated for uveal melanoma, including patient demographics, tumour characteristics (including histology and tumour location), primary treatment, further treatments (including enucleation and vitrectomies), starting and final visual acuities, and mortality. This allowed for the identification of patients with uveal melanoma who underwent vitrectomy due to complications of their treatment between 2012 and 2022 using the following inclusion criteria: confirmed diagnosis of uveal melanoma of the choroid or ciliary body, first presentation between 1 January 2021 and 31 December 2022, primary treatment undergone at the LOOC, and primary treatment of either proton-beam radiotherapy or plaque brachytherapy. All patients who received plaque brachytherapy at LOOC received Ru-106 plaque therapy. The standard treatment dose for plaque brachytherapy was a minimum of 90 Gy to the apex and assuming an appropriate distance from the optic disc and fovea a maximum of 350 Gy; however, actual doses vary according to tumour thickness and location. The standard treatment dose for proton-beam radiotherapy was a 52 Gy total radiation dose given to the patient.

**Figure 1:**
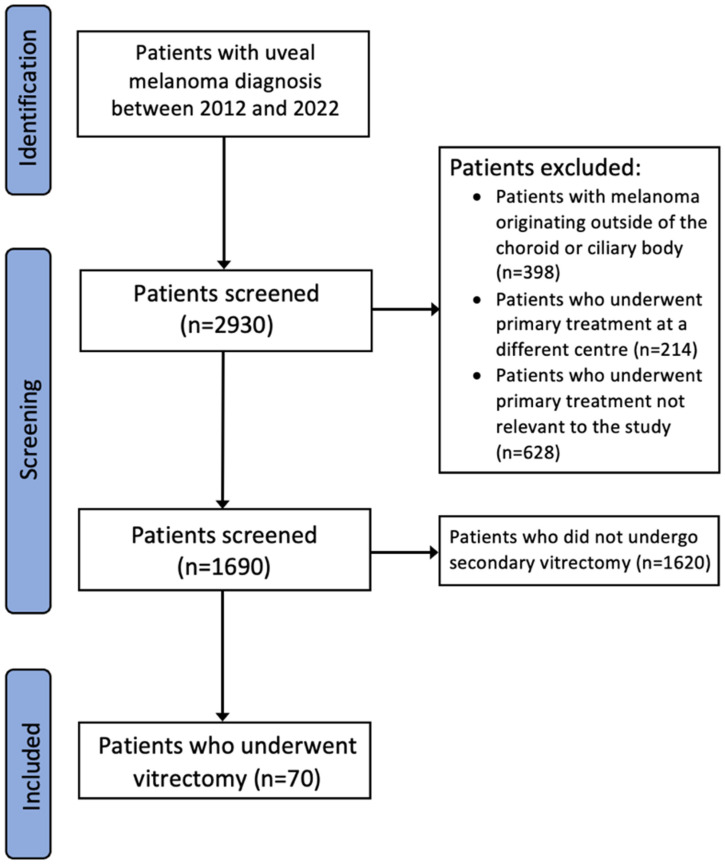
Diagram showing patient selection process.

Exclusion criteria included tumours originating in the iris, conjunctiva, retina, caruncle, canthus, and retinal pigment epithelium (*n* = 398). Melanomas which underwent primary treatments at a different hospital or that were not relevant to the study were excluded, including enucleation, observation, biopsy alone, transpupillary thermotherapy, photodynamic therapy, endoresection, local resection, or combined endoresection and plaque (*n* = 842). The exclusion process is highlighted in Figure 1.

Ethical review and approval were waived for this study as this is an approved registered audit (AMaT Project Code: Ophth/SE/2025-26/19) with anonymised retrospective data which did not influence standard-of-care decisions.

Where available, patient records were extracted manually for indication for vitrectomy, time from diagnosing the complication to the date of vitrectomy, serial visual acuities, indication for enucleation, tumour recurrence, and metastatic spread. Outcome measures included overall tumour control, visual outcome, and metastatic mortality.

All visual acuities were converted from Snellen to LogMAR for analysis. Counting of fingers was converted to LogMAR 2.10, hand movement was converted to LogMAR 2.40, light perception was converted to LogMAR 2.70, and no light perception was converted to LogMAR 3.00 (all assumed a distance of one meter) [26]. Where possible, visual acuities were collected at the most recent pre-vitrectomy date before the indication for vitrectomy was present, and at the closest date to 1-, 2-, 3-, and 4-years post-vitrectomy as well as at final follow-up. Data was excluded if there was inadequate record retrieval.

The association of vitrectomy with metastatic death, observed local recurrence, and enucleation was analysed. Due to the presence of death events, a Kaplan–Meier analysis would not be appropriate as the results would be biased and possibly nonsensical; therefore, the Aalen–Johansen (AJ) estimator [27] was used for graphical assessment as it allows for estimation of the probability of the events of interest in the presence of competing risks. For metastatic death, deaths due to other causes were assumed as a competing risk; for observed local recurrence and enucleation, any death was assumed as a competing risk. Cox analysis was also used to estimate the effect of vitrectomy on hazard rates for the same outcomes. Although the Cox model is not directly affected by the issue of competing risks, it should be noted that vitrectomy is a time-dependent covariate [27], since it is not observed at tumour management but at different subject-specific times post management; this requires a special formulation of Cox analysis using counting process techniques [27].

A *p*-value of <0.05 was considered statistically significant.

## 3. Results

Data was extracted from the patient records of the 1690 patients that met the inclusion criteria. The groups were divided into those who had primary radiotherapy with unplanned secondary vitrectomy (*n* = 70) and the remainder of the patients (*n* = 1620).

The mean age was similar between the groups (58.0 years, range: 28 years–90 years versus 61.1 years, range: 10 years–93 years), while tumour characteristics differed greatly; the vitrectomy group had a higher proportion of tumours originated from the ciliary body (10/70 patients, 14.3% versus 142/1620 patients, 8.8%), tumour dimensions were larger (mean basal diameter 12.05 mm, range: 3.3 mm–18.7 mm vs. 8.82 mm, range: 1.8 mm–18.3 mm; mean thickness 4.86 mm, range: 0.9 mm–11.8 mm vs. 3.07 mm, range: 0.3 mm–13.8 mm), and there was a higher rate of monosomy 3 (21/49 patients, 42.9% versus 217/602 patients, 36.0%). Those who underwent primary proton-beam radiotherapy were more likely to undergo secondary vitrectomy than plaque brachytherapy patients (45/844 patients, 5.3% versus 25/846 patients, 3.0%) (Table 1 and File S1).

### 3.1. Indication for Vitrectomy

The most common indications for unplanned secondary vitrectomy (*n* = 70) were vitreous haemorrhage (27/70 patients, 38.6%) and exudative retinal detachment/toxic tumour syndrome (22/70 patients, 31.4%). In the proton-beam radiotherapy group, the same two indications of vitreous haemorrhage (18/45 patients, 40.0%) and exudative retinal detachment/TTS (17/45 patients, 37.8%) predominate, while in the plaque brachytherapy group the commonest indications were more varied including vitreous haemorrhage (9/25 patients, 36.0%), epiretinal membrane (5/25 patients, 20.0%), exudative retinal detachment/TTS (5/25 patients, 20.0%), and rhegmatogenous retinal detachment (4/25 patients, 16.0%) (Table 2 and Table 3, and File S1).

### 3.2. Revisional Vitreoretinal Procedures

Additional revision vitreoretinal procedures were required after secondary vitrectomy in twelve eyes (12/70 patients, 17.1%); eight patients underwent initial proton-beam radiotherapy and four patients underwent plaque brachytherapy. Four were indicated for exudative retinal detachment (4/12, 33.3%), two for rhegmatogenous retinal detachment (2/12, 16.7%), five for vitreous haemorrhage (5/12, 41.7%), and one for macular hole (1/12, 8.3%).

Secondary treatments were required in thirteen eyes (13/70 patients, 18.6%) after secondary vitrectomy, with one eye requiring three follow-up treatments (1/13 patient, 7.7%), two eyes requiring two follow-up treatments (2/13 patients, 15.4%), and ten eyes requiring one follow-up treatment (10/13 patients, 76.9%). These treatments included six enucleations, four transpupillary thermotherapy procedures, two cryotherapy procedures, two endoresections, and three photocoagulation procedures.

### 3.3. Patient Co-Morbidities

Co-morbidities were recorded at initial presentation with the most common conditions listed as hypertension, diabetes, ischaemic heart disease, and other malignancies. The proton-beam group had a higher proportion of hypertension (11/45 patients, 24% versus 2/25 patients, 8%) while the plaque brachytherapy group had a higher proportion of diabetes (3/25 patients, 12% versus 1/45 patient, 2%). Similar incidences were reported of ischaemic heart disease (3/45 proton-beam patients, 7% versus 2/25 plaque brachytherapy patients, 8%) and other malignancies (5/45 proton-beam patients, 11% versus 2/25 plaque brachytherapy patients, 8%). Other co-morbidities in the plaque brachytherapy group included TIA (1/25 patient, 4%), asthma (1/25 patient, 4%), Paget’s disease (1/25 patient, 4%), and bipolar disorder (1/25 patient, 4%), while in the proton-beam radiotherapy group additional co-morbidities included asthma (2/45 patients, 4%), Crohn’s disease (1/45 patient, 2%), and chronic kidney disease (1/45 patient, 2%).

### 3.4. Tumour Control

Rates of local recurrence were lower in the vitrectomy group (1/70 patient, 1.4% versus 30/1620 patients, 1.9%). In those who underwent vitrectomy (*n* = 70), proton-beam radiotherapy patients had higher rates of local recurrence compared to plaque brachytherapy patients (1/45 patient, 2.2% versus 0/25 patients, 0.0%).

In radiotherapy patients who did not undergo vitrectomy, global tumour control was poorer in the proton-beam radiotherapy primary treatment group than the plaque brachytherapy treatment group with a local recurrence rate of 2.5% (20/799 patients, 2.5% versus 10/821 patients, 1.2%).

**Figure 2:**
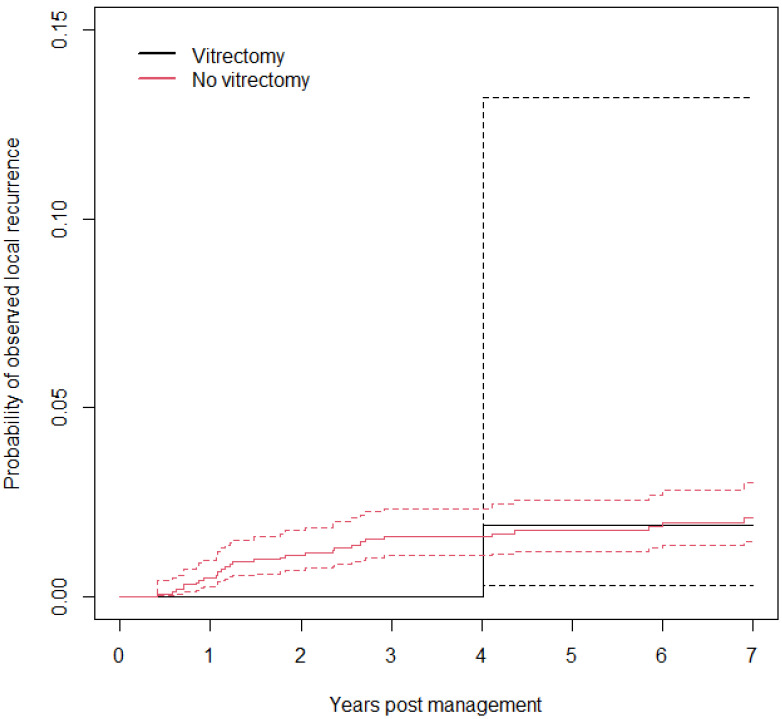
Probability of observed local recurrence by vitrectomy with a 95% confidence interval.

The AJ estimates of the probability of observed local recurrence by vitrectomy are shown in Figure 2. It is not possible to infer significant results, since only one patient who underwent vitrectomy developed local recurrence.

### 3.5. Eye Retention

Enucleation rates were higher in patients who underwent secondary vitrectomy (6/70 patients, 8.6% versus 43/1620 patients, 2.7%) in keeping with their larger tumour size. Proton-beam radiotherapy patients had higher rates of enucleation compared to plaque brachytherapy patients (5/45 patients, 11.1% versus 1/25 patients, 4.0%). The mean interval from first visit to enucleation was 2.80 years (range: 1.32 years–5.33 years); the commonest reason for enucleation was rubeotic glaucoma in three patients (3/6 patients, 50.0%) followed by melanoma recurrence in one patient (1/6 patient, 16.7%), toxic tumour syndrome in one patient (1/6 patient, 16.7%), and expulsive vitreous haemorrhage in one patient (1/6 patient, 16.7%).

**Figure 3:**
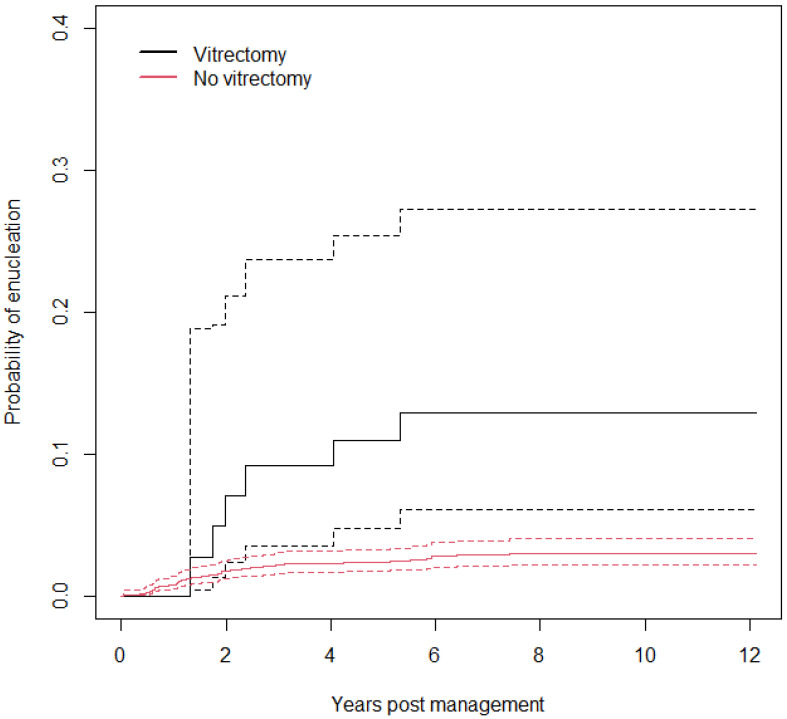
Probability of enucleation by vitrectomy with a 95% confidence interval.

The AJ estimates of the probability of enucleation by vitrectomy are shown in Figure 3. Although the estimates are well-separated, due to the small number of events, the confidence intervals for the group who underwent vitrectomy are extremely wide (only 6/70 patients who underwent vitrectomy also underwent enucleation).

Cox analysis shows that the hazard ratio for vitrectomy is 5.7 [2.5, 13], with a robust score statistic *χ*^2^ = 4.2 (*p* = 0.04), denoting little evidence of association of vitrectomy with enucleation. The Schoenfeld test for proportionality of hazards (PH) is *χ*^2^ = 0.00001 (*p* = 0.997); thus, there is no evidence of the violation of the PH assumption of the Cox model. The C index is 0.55 [0.50, 0.59], denoting poor rank discrimination.

### 3.6. Metastatic Mortality

Metastatic incidence was higher in patients who underwent secondary vitrectomy (11/70 patients, 15.7% versus 91/1620 patients, 5.6%) in keeping with their larger tumour size and higher rates of monosomy 3, with approximately the same proportion of patients in the proton-beam radiotherapy and plaque brachytherapy groups (7/45 patients, 15.6% versus 4/25 patients, 16.0%); a total of eight patients (8/11 patients, 72.7%) initially presenting with a liver lesion and three patients (3/11 patients, 27.3%) initially presenting with a lung lesion, occurring at a mean interval from first visit of 2.96 years (range: 0.28 years–6.48 years). It was later found that metastasis occurred before vitrectomy in two patients, meaning that the total number of patients who were diagnosed with metastases after secondary vitrectomy was nine (9/70 patients, 12.9%), at a mean interval of 3.50 years (range: 1.18 years–6.48 years), with three patients (3/70 patients, 4.3%) diagnosed with metastases by three years, and seven patients (7/70 patients, 10.0%) diagnosed with metastases by five years.

In addition, all-cause mortality (8/70 patients, 11.4% versus 170/1620 patients, 10.5%) and metastatic mortality (6/70 patients, 8.6% versus 41/1620 patient, 2.5%) were higher in the secondary vitrectomy group. The all-cause mortality rates of post-vitrectomy proton-beam radiotherapy patients were higher than those of plaque radiotherapy patients (7/45 patients, 15.6% versus 1/25 patient, 4.0%); this also applied to metastatic mortality rates (5/45 patients, 11.1% versus 1/25 patient, 4.0%) (Table 4 and Table 5, and File S1).

**Figure 4:**
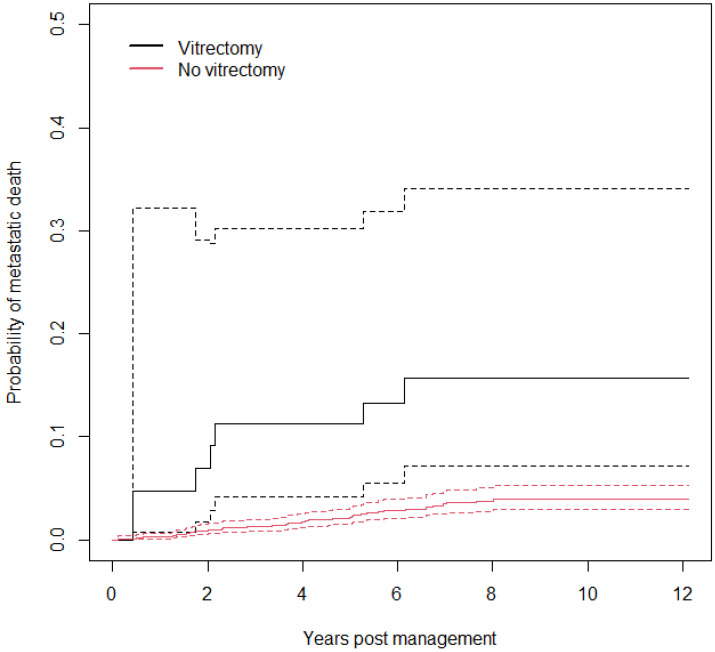
Probability of metastatic death by vitrectomy with a 95% confidence interval.

The AJ estimates of the probability of metastatic death by vitrectomy are shown in Figure 4. Although the estimates are well-separated, due to the small number of events the confidence intervals for the group which underwent vitrectomy are extremely wide (only 6/70 patients who underwent vitrectomy died due to metastases).

Cox analysis shows that the hazard ratio for vitrectomy is 4.9 [1.6, 9.5], with robust score statistic *χ*^2^ = 3.3 (*p* = 0.07), denoting little evidence of association of vitrectomy with metastatic death. The Schoenfeld test for proportionality of hazards (PH) is *χ*^2^ = 2.1 (*p* = 0.2); thus, there is no evidence of the violation of the PH assumption of the Cox model. The C index is 0.55 [0.50, 0.59], denoting poor rank discrimination.

### 3.7. Visual Acuity

Visual acuities were examined in secondary vitrectomy radiotherapy patients. The mean visual acuity for the entire cohort at the most recent pre-vitrectomy date within our database was 0.60 LogMAR. In our cohort (excluding artificial eyes), visual acuities improved by three lines or more on a Snellen chart in 14.1% (9/64 patients, 14.1%) of patients from their pre-vitrectomy measurements and the measurement at final follow-up. Plaque brachytherapy had a higher proportion of eyes with an improvement of three lines or greater compared to proton-beam patients (4/24 patients, 16.7% versus 5/40 patients, 12.5%), while 25.0% (16/64 patients, 25.0%) of patients experienced any degree of visual improvement. Plaque brachytherapy patients had a higher proportion of eyes with improvement compared to proton-beam patients (9/24 patients, 37.5% versus 7/40 patients, 17.5%). A visual decline of three lines or greater was seen in 50.0% (32/64 patients, 50.0%) of patients; proton-beam radiotherapy patients had a higher proportion of eyes with this degree of decline compared to plaque brachytherapy patients (25/40 patients, 62.5% versus 7/24 patients, 29.2%).

The indications for vitrectomy of the patients with an improvement of three lines or greater at final follow-up were exudative retinal detachment (3/22 patients, 13.6%), rhegmatogenous retinal detachment (1/7 patient, 14.3%), epiretinal membrane (1/7 patient, 14.3%), vitreous haemorrhage (2/27 patients, 7.4%), macular hole (1/1 patient, 100.0%), and endophthalmitis (1/1 patient, 100.0%).

At final follow-up, the mean visual acuity decline after secondary vitrectomy was 0.55 LogMAR, with measurements ranging from a 2.30 LogMAR improvement to a 3.06 LogMAR decline. Mean visual acuity declined in both treatment groups, with proton-beam radiotherapy having the most significant decline (mean 0.78 LogMAR decline versus 0.17 LogMAR decline). When looking at the visual acuity change in secondary vitrectomy patients at various time intervals from the date of vitrectomy, we see a generalised decline from the most recent pre-vitrectomy measurement (1 year: 0.40 LogMAR decline; 2 years: 0.47 LogMAR decline; 3 years: 0.60 LogMAR decline; 4 years: 0.65 LogMAR decline; final follow-up: 0.55 LogMAR decline).

When comparing visual outcomes in patients based on the indication for vitrectomy, there is little difference in the mean decline at final follow-up between groups. The decline in LogMAR acuity at final follow-up for vitreous haemorrhage was a 0.54 LogMAR decline (*n* = 27, range: 2.30 LogMAR improvement to 3.00 LogMAR decline), for exudative retinal detachment/toxic tumour syndrome there was a 0.65 LogMAR decline (*n* = 22, range: 0.6 LogMAR improvement to 3.06 LogMAR decline), for rhegmatogenous retinal detachment there was a 0.62 LogMAR decline (*n* = 7, range: 0.5 LogMAR improvement to 2.3 LogMAR decline), for ERM there was a 0.30 LogMAR decline (*n* = 16, range: 0.5 LogMAR improvement to 1.7 LogMAR decline), and for pigmented vitreous infiltrates there was a 0.75 LogMAR decline (*n* = 4, range: 0.2 LogMAR decline to 1.5 LogMAR decline) (Table 6 and File S1).

Patients requiring vitrectomy for exudative retinal detachment (*n* = 22) had generally poorer outcomes with high rates of enucleation (5/22 patients, 22.7%), metastatic disease (5/22 patients, 22.7%), and all-cause mortality (3/22 patients, 13.6%); this is likely due to the larger size of these tumours (mean tumour diameter: 13.89 mm, mean tumour thickness: 6.27 mm). After vitrectomy for rhegmatogenous retinal detachment, no patients underwent enucleation procedures or were diagnosed with local recurrence or metastases (Table 7 and File S1).

### 3.8. Retinal Detachment

In total, 29 patients underwent secondary vitrectomy for retinal detachment, 7 for rhegmatogenous detachment (7/29 patients, 24.1%), and 21 for exudative retinal detachment not including 1 exclusive toxic tumour syndrome patient (22/29 patients, 75.9%). Adequate record retrieval was not possible for one patient within this cohort. Of the 23 patients who had oil insertion, 11 patients never had oil removal (11/23 patients, 47.8%), 10 patients (10/21 patients, 47.6%) after exudative retinal detachment, and 1 patient (1/7 patients, 14.3%) after rhegmatogenous retinal detachment. Of the retinal detachment patients who never had oil removal, nine patients underwent initial proton-beam radiotherapy (9/19 patients, 47.4% of proton-beam patients with retinal detachment) and two patients underwent initial plaque brachytherapy (2/9 patients, 22.2% of plaque patients with retinal detachment).

Six patients underwent additional vitreoretinal procedures to achieve a flat retina, with four patients undergoing a single additional procedure (rhegmatogenous *n* = 1, exudative *n* = 3) and two patients undergoing two additional procedures (exudative *n* = 2). Of the patients who underwent these further procedures, five patients had initial proton-beam radiotherapy (5/19 patients, 26.3% of proton-beam patients with retinal detachment) and one patient had initial plaque brachytherapy (1/9 patients, 11.1% of plaque patients with retinal detachment). Both patients who required two additional procedures underwent initial plaque brachytherapy.

In five patients, a ‘flat retina’ was never achieved (5/28, 17.9%). All of these patients underwent primary proton-beam radiotherapy (5/19, 26.3% of all proton-beam patients with retinal detachment), and the oil was never removed. All patients achieved a ‘flat retina’ in the plaque radiotherapy group, of whom all except two patients had oil removal.

## 4. Discussion

Complications after globe-sparing uveal melanoma treatment are uncommon but may require swift vitreoretinal intervention to maximise patient outcomes. Vitrectomy may be utilised to manage such complications. Some interventions aim to improve visual outcomes, while others aim to avoid enucleation or to improve evaluation of the tumour treatment response. Our study from 2012 to 2022 showed that 70/1690 (4.1%) patients underwent vitrectomy after radiotherapy treatment for uveal melanoma, which explains why the evidence base is weak in single-centre studies around the topic.

The highest rates of posterior segment intervention were seen following proton-beam radiotherapy patients (5.3%) versus plaque brachytherapy patients (3.0%). The mean age at diagnosis of secondary vitrectomy patients was slightly lower than in the rest of the cohort (58.0 years versus 61.1 years). It is likely that younger patients will be more driven to undergo repeated surgical procedures to retain their eye following the development of complications rather than opt for enucleation. In addition, proton beam treatment usually entails lower radiation energies and wider radiation fields compared to Ruthenium plaque radiotherapy. In daily practice, the regression of tumours is slower following proton therapy, and there is more destruction of the affected and the surrounding choroidal and retinal tissue leading to an increased vasoproliferative drive, as well as an increase in the level of toxic tumour breakdown with haemorrhagic and exudative complications.

It is important to acknowledge that certain complications of primary treatment for uveal melanoma proliferative retinopathy or exudative retinal detachment may be amenable to medical therapy such as laser therapy or intravitreal injections including steroids and anti-VEGF [5,13,28]. However, in our cohort these options would have been attempted prior to undertaking vitreoretinal surgery if indicated and possible.

Statistical analysis demonstrates that secondary vitrectomy had a statistically significant impact on enucleation rates (*p* = 0.04) in keeping with the larger tumour sizes; it is important to note that due to the small numbers in the vitrectomy group, no adjustment for tumour characteristics was possible. Despite this, there was no statistically significant impact on the incidence of metastatic death (*p* = 0.07). Interestingly, local recurrence rates were lower in secondary vitrectomy patients.

Proton-beam radiotherapy with subsequent secondary vitrectomy was associated with higher rates of enucleation (11.1% versus 4.0%), local recurrence (2.2% versus 0.0%), all-cause mortality (15.6% versus 4.0%), and metastatic mortality (11.1% versus 4.0%) compared to plaque treatment. This is likely because tumours treated with proton-beam radiotherapy tend to be larger than those treated with plaque brachytherapy; however, due to small sample size we were unable to control for tumour size in this study. The prevalence of certain co-morbidities such as hypertension (24% versus 8%) and diabetes (2% versus 12%) varied between the proton-beam radiotherapy and plaque brachytherapy patient groups who underwent secondary vitrectomy; however, given the small sample sizes and multiple confounding factors such as age and time of presentation this is unlikely to impact outcomes significantly.

The most common indication for enucleation after primary radiotherapy and secondary vitrectomy was neovascular glaucoma (3/6 patients, 50.0% of cases); this is thought to be due to radiotherapy-induced effects such as vasculopathy, ischaemia, and neovascularisation. Secondary enucleation rates after vitrectomy in uveal melanoma vary greatly between studies. In a case series of three patients with choroidal melanoma who underwent vitrectomy [29], two patients (2/3 patients, 66.7%) underwent enucleation; Bansal et al. [20] report that enucleation was required in 6 of 47 eyes (6/47 patients, 12.8%) after plaque brachytherapy and subsequent vitrectomy. Further reporting by Chia et al. [4] noted that in 10 eyes, none required enucleation (0/10 patients, 0.0%). These rates will be heavily dependent on the indication for vitrectomy and the severity of the disease complications, related to the extent of the tumour and dose/methodology of primary treatment modality.

Retinal detachment is a less common indication for vitrectomy post-uveal melanoma treatment. Grixti et al. [30] reported five patients who experienced rhegmatogenous retinal detachment requiring vitrectomy after primary melanoma treatment, with only two patients (2/5 patients, 40.0%) achieving a flat retina after the first vitrectomy. On the contrary, Beykin et al. [31] reported that seven of seven patients who had a retinal detachment following plaque brachytherapy for uveal melanoma had retinas that were attached at the final date of follow-up (mean duration 18.4 months), with five of the seven patients experiencing substantial improvement in vision. Of these seven patients, three had combined tractional and rhegmatogenous retinal detachment, three had rhegmatogenous retinal detachment, and one had a tractional retinal detachment. Our series has shown a successful retinal reattachment rate of 85.7% (6/7 patients, 85.7%) in rhegmatogenous and 47.6% (10/21 patients, 47.6%) in exudative cases after single surgery. The rates reflect the complex nature of these eyes and the associated poor state of the irradiated retina.

Visual outcomes will be variable according to the indication for vitrectomy and the extent/location of the tumour. Visual acuities post-secondary vitrectomy have been reported to improve in patients who undergo initial proton-beam radiotherapy, suggesting a mean improvement at 22 to 23 months follow-up post-vitrectomy of 0.30 LogMAR [9]; the most common indications for vitrectomy were vitreous haemorrhage (*n* = 13) and epimacular membrane (*n* = 5), although no comparison in visual improvement was made between indications. The reported outcomes after plaque radiotherapy are more variable. Lonngi et al. [32] showed a mean improvement of 0.36 LogMAR at one year post-secondary vitrectomy, with vitrectomy performed for radiation-related complications including retinal detachment (*n* = 102), vasculopathy (*n* = 91), optic neuropathy (*n* = 32), and vitreous haemorrhage (*n* = 8); patients could have one or more contributing factors for the decision for vitrectomy. A case-series by Bansal et al. [20] investigated the visual acuities of 47 patients that underwent secondary vitrectomy for vitreous haemorrhage after plaque radiotherapy, where prior to vitrectomy 91.5% (43/47 patients, 91.5%) of patients were seeing 1.00 LogMAR or worse, which improved to 72.3% (34/47 patients, 72.3%) seeing 1.00 LogMAR or worse after vitrectomy. McCannel et al. [33], however, report worsened visual outcomes in patients treated with vitrectomy following plaque brachytherapy, with a mean decline of 0.70 LogMAR and 65.0% (13/20 patients, 65.0%) of eyes seeing better than 1.00 LogMAR at final follow-up. In our post-radiotherapy secondary vitrectomy patients, there was a mean decline of 0.55 LogMAR from pre-vitrectomy baseline to final follow-up, likely due to the posterior location of tumours treated with proton-beam radiotherapy affecting the optic nerve and macular function. However, there was great variation between patients (range: 2.30 LogMAR improvement–3.06 LogMAR decline) reflecting the variable tumour sizes, radiation doses, location, and subsequent complications indicating the need for vitrectomy.

Multiple studies within the literature have documented the visual outcomes following plaque brachytherapy and proton-beam radiotherapy, obviously with proximity to the fovea and disc being the most predictive; however, tumour volume also has an important influence [34,35,36,37,38]. This is key to the potential of exudative complications as well as the development of vasculopathy due to the wide area of the treated retina.

A multidisciplinary approach to patient care involving vitreoretinal surgeons and ocular oncologists enables early recognition and management of complications following uveal melanoma treatment; however, it is prudent to ensure that the tumour is controlled before vitreoretinal intervention is undertaken to reduce the risk of intraocular and extraocular spread [19,22,39]. So-called ‘Onco-VR surgeons’ could play a vital role in maximising functional outcomes for patients with treatment-related complications requiring vitrectomy [39]. The rarity of such cases and the variability of cohorts makes it difficult to compare studies. This is the largest study to assess the incidence, indications, and outcomes of uveal melanoma patients undergoing secondary vitrectomy for treatment-associated complications.

A major strength of this study is that it is the largest cohort of its kind with over ten years of experience in a unit manned by Onco-VR specialists. Some of the weaknesses include the study’s retrospective design and the fact that it only accounts for patients who underwent surgical intervention and not those who underwent enucleation once complications had developed.

## 5. Conclusions

Radiotherapy is an important treatment in the management of uveal melanoma; vitreoretinal complications are uncommon but remain a risk of uveal melanoma treatment. Primary proton therapy is more likely to produce exudative complications. Vitreoretinal surgeons and ocular oncologists play a crucial role in managing and achieving optimal patient outcomes and preserving more eyes.

## Figures and Tables

**Table 1:** Tumour characteristics per primary treatment group.

	Treatment Group	No Vitrectomy	Vitrectomy
Number of patients	Total (*n* = 1690)	1620/1690 (95.9%)	70/1690 (4.1%)
	PBRT ^1^ (*n* = 844)	799/844 (94.7%)	45/844 (5.3%)
	Ru-106 PB ^2^ (*n* = 846)	821/846 (97.0%)	25/846 (3.0%)
Mean age (years)	Total (*n* = 1690)	61.1 (range: 10–93)	58.0 (range: 28–90)
	PBRT ^1^ (*n* = 844)	59.0 (range: 10–93)	54.2 (range: 28–82)
	Ru-106 PB ^2^ (*n* = 846)	63.2 (range: 16–92)	64.7 (range: 31–90)
Mean LBD ^3^ (mm)	Total (*n* = 1690)	8.82 (range: 1.8–18.3)	12.1 (range: 3.3–18.7)
	PBRT ^1^ (*n* = 844)	10.2 (range: 1.4–22.6)	12.0 (range: 3.3–17.7)
	Ru-106 PB ^2^ (*n* = 846)	10.3 (range: 3.0–19.0)	12.1 (range: 6.7–18.7)
Mean thickness (mm)	Total (*n* = 1690)	3.07 (range: 0.3–13.8)	4.86 (range: 0.9–11.8)
	PBRT ^1^ (*n* = 844)	3.1 (range: 0.3–13.8)	5.4 (range: 0.9–11.8)
	Ru-106 PB ^2^ (*n* = 846)	3.0 (range: 0.4–10.9)	4.0 (range: 1.7–6.8)
Monosomy 3 presence	Total (*n* = 1690)	217/602 (36.0%)	21/49 (42.9%)
	PBRT ^1^ (*n* = 844)	114/287 (39.7%)	16/32 (50.0%)
	Ru-106 PB ^2^ (*n* = 846)	103/315 (32.7%)	5/17 (29.4%)

Percentages of monosomy 3 take into account only those with a pathology result. ^1^ Proton-beam radiotherapy. ^2^ Plaque brachytherapy. ^3^ Longitudinal basal diameter.

**Table 2:** Number of patients treated per indication.

Indication	Number of Patients (%)	Mean Tumour Diameter (mm)	Mean Tumour Thickness (mm)
Vitreous haemorrhage	27 (38.6%)	10.93 (range: 4.0–17.7)	4.19 (range: 1.0–9.9)
Exudative RD ^1^/TTS ^2^	22 (31.4%)	13.89 (range: 9.3–18.7)	6.27 (range: 1.3–11.8)
Epiretinal membrane	7 (10.0%)	11.24 (range: 6.7–16.6)	4.50 (range: 1.7–6.8)
Rhegmatogenous RD ^1^	7 (10.0%)	11.97 (range: 10.2–16.2)	3.93 (range: 2.2–5.5)
Pigmented vitreous infiltrates	4 (5.7%)	14.40 (range: 9.5–17.1)	6.45 (range: 2.7–11.3)
Macular hole	1 (1.4%)	10	2.1
Neovascular glaucoma	1 (1.4%)	3.3	0.9
Endophthalmitis	1 (1.4%)	9.9	1.2

^1^ Retinal detachment. ^2^ Toxic tumour syndrome.

**Table 3:** Indication for vitrectomy per treatment group.

Primary Treatment	Proportion of Patients Undergoing Vitrectomy	Indication	Value (%)	Proportion of Patients Undergoing Vitrectomy per Initial Treatment
Proton-beam radiotherapy (*n* = 45)	45/844 (5.3%)	Exudative RD ^1^/TTS ^2^	17 (37.8%)	17/844 (2.0%)
		Vitreous haemorrhage	18 (40.0%)	18/844 (2.1%)
		Rhegmatogenous RD ^1^	3 (6.7%)	3/844 (0.4%)
		Epiretinal membrane	2 (4.4%)	2/844 (0.2%)
Ru-106 plaque brachytherapy (*n* = 25)	25/846 (3.0%)	Exudative RD ^1^/TTS ^2^	5 (20.0%)	5/846 (0.6%)
		Vitreous haemorrhage	9 (36.0%)	9/846 (1.1%)
		Rhegmatogenous RD ^1^	4 (16.0%)	4/846 (0.5%)
		Epiretinal membrane	5 (20.0%)	5/846 (0.6%)

^1^ Retinal detachment. ^2^ Toxic tumour syndrome.

**Table 4:** Secondary effects of patients treated with primary radiotherapy.

	Treatment Group	
	Vitrectomy (*n* = 70)	Non-Vitrectomy (*n* = 1620)
Enucleation	6 (8.5%)	43 (2.7%)
Local recurrence	1 (1.4%)	30 (1.9%)
Metastases	11 (15.7%)	91 (5.6%)
Metastatic mortality	6 (8.6%)	46 (2.8%)

Values are presented as number of patients (%).

**Table 5:** Secondary effects of patients treated with primary radiotherapy per treatment group.

	Primary Treatment			
	Proton-Beam Radiotherapy		Plaque Brachytherapy	
	NV ^1^ (*n* = 799)	V ^2^ (*n* = 45)	NV ^1^ (*n* = 821)	V ^2^ (*n* = 25)
Enucleation	37 (4.6%)	5 (11.1%)	6 (0.7%)	1 (4.0%)
Local recurrence	20 (2.5%)	1 (2.2%)	10 (1.2%)	0 (0.0%)
Metastases	57 (7.1%)	7 (15.6%)	34 (4.1%)	4 (16.0%)
Mortality	83 (10.4%)	7 (15.6%)	87 (10.6%)	1 (4.0%)
Metastatic mortality	28 (3.5%)	5 (11.1%)	13 (1.6%)	1 (4.0%)

Values are presented as number of patients (%). ^1^ Non-vitrectomy group. ^2^ Vitrectomy group.

**Table 6:** Mean visual acuity per indication for vitrectomy; comparison of pre-PPV acuity and post-PPV acuity.

Indication	Overall Change	Final Follow-Up Mean Change in Visual Acuity (LogMAR)
Vitreous haemorrhage	Declined	0.54 (2.30 improvement–3.00 decline)
Exudative RD ^1^/TTS ^2^	Declined	0.65 (0.60 improvement–3.06 decline)
Epiretinal membrane	Declined	0.30 (0.50 improvement–1.70 decline)
Rhegmatogenous RD ^1^	Declined	0.62 (0.50 improvement–2.30 decline)
Pigmented vitreous infiltrates	Declined	0.75 (0.20 decline–1.50 decline)
Macular hole	Improved	−0.38
Neovascular glaucoma	Artificial eye	Artificial eye
Endophthalmitis	Declined	1.00

Values are presented as change in LogMAR visual acuity (range). ^1^ Retinal detachment. ^2^ Toxic tumour syndrome.

**Table 7:** Outcome rates per indication for secondary vitrectomy.

Indication	Enucleation Rate	Local Recurrence	Metastases
Vitreous haemorrhage (*n* = 27)	0/27 (0.0%)	0/27 (0.0%)	4/27 (14.8%)
Exudative RD ^1^/TTS ^2^ (*n* = 22)	5/22 (22.7%)	1/22 (4.5%)	5/22 (22.7%)
Epiretinal membrane (*n* = 7)	0/7 (0.0%)	0/7 (0.0%)	1/7 (14.3%)
Rhegmatogenous RD ^1^ (*n* = 7)	0/7 (0.0%)	0/7 (0.0%)	0/7 (0.0%)
Pigmented vitreous infiltrates (*n* = 4)	0/4 (0.0%)	0/4 (0.0%)	1/4 (25.0%)
Macular hole (*n* = 1)	0/1 (0.0%)	0/1 (0.0%)	0/1 (0.0%)
Neovascular glaucoma (*n* = 1)	1/1 (100.0%)	0/1 (0.0%)	0/1 (0.0%)
Endophthalmitis (*n* = 1)	0/1 (0.0%)	0/1 (0.0%)	0/1 (0.0%)

Values are presented as number of patients (%). ^1^ Retinal detachment. ^2^ Toxic tumour syndrome.

## Data Availability

Raw data is available to view in the supplementary documents.

## Supplementary Materials

The following supporting information can be downloaded at https://www.mdpi.com/article/10.3390/cancers18010095/s1, File S1: Vitreoretinal surgery for intraocular complications following radiotherapy treatment of uveal melanoma: raw data file.
